# Analysis of Piano Performance Characteristics by Deep Learning and Artificial Intelligence and Its Application in Piano Teaching

**DOI:** 10.3389/fpsyg.2021.751406

**Published:** 2022-01-27

**Authors:** Weiyan Li

**Affiliations:** School of Music, Southwest University, Chongqing, China

**Keywords:** intelligent piano, functional characteristics, preschool children, enlightenment teaching, smart piano

## Abstract

Deep learning (DL) and artificial intelligence (AI) are jointly applied to concrete piano teaching for children to comprehensively promote modern piano teaching and improve the overall teaching quality. First, the teaching environment and the functions of the intelligent piano are expounded. Then, a piano note onset detection method is proposed based on the convolution neural network (CNN). The network can analyze the time-frequency of the input piano music signal by transforming the original time-domain waveform of the piano music signal into the frequency distribution varying with time. Besides, it can detect the note onset at a stable state after 8 × 10^4^ iterations. Moreover, an intelligent piano teaching method is designed to teach *Jingle Bells* for 40 preschool children aged 4–6 years. Finally, a questionnaire survey is performed to investigate the teaching situation, including the learning interest and learning effect of children and learning feedback from parents. The results show that 80% of children like smart music scores, 82% of children like intelligent piano lessons with games, and 84% of children can learn actively in the intelligent piano class. Besides, 85% of parents believe that their children are more interested in learning piano. In general, the intelligent piano teaching method effectively combines DL with AI to realize the overall optimization of piano performance. It is widely favored by preschool children and their parents and plays an important role in improving the interest of preschool children in piano learning.

## Introduction

With the high-speed economic development and the continuous improvement of living standards of residents in China, parents pay increasingly more attention to the comprehensive development of preschool children. Mounting preschool children are choosing to learn intelligent piano, especially in developed cities (Wahl et al., 2021). The intelligent piano can be connected to intelligent devices through the Internet to teach learners online, gradually realizing the intellectualization of the piano (Williams et al., 2017). Since the intelligent piano “The One” came out in 2015 and has been used in piano teaching, there is a proliferation of intelligent piano training classes nationwide. The mechanical principle of the traditional piano can be combined with Internet information technology (IT), automatic control technology, and artificial intelligence (AI) technology to form the intelligent piano. With the development of science and technology, the intelligent piano comes with its teaching software. Users can connect the intelligent piano to an add-on program (on mobile phone or iPad) using the Internet, and they can easily learn piano with advanced games. The main enrollment object of intelligent piano teaching is preschool children. Piano teaching for preschool children belongs to the stage of piano enlightenment education (Androsova, 2017). How can intelligent piano promote preschool children? How to let preschool children learn playing piano? These are the problems that need to be considered in the teaching of intelligent piano for preschool children.

The current school music curriculum can fully apply the means of science and technology. According to the change in the communication mode between teachers and students in the music class, the fit between their music classroom situation and different social cultures is also undergoing subtle changes. On the one hand, big data analysis can enable school managers to find loopholes in the existing teaching system and further optimize the teaching structure. On the other hand, smart education has brought novel teaching methods for teachers and students, such as intelligent piano, and diverse music learning software has changed the habits of music learners. Moreover, deep learning (DL) can be applied to intelligent piano teaching. For example, the automatic music transcription method based on DL can compare played music with the standard score to automatically and objectively justify the correctness of performance, to help learners find their errors in time, and to improve learning efficiency. This technology can be applied to computer-aided piano teaching and piano grade examinations.

In this study, the first section sorts the research relevant to intelligent music teaching. The second section expatiates the function and characteristics of intelligent piano, combining with the physical and mental development of preschool children. Based on this, a convolution neural network (CNN) is employed to design a piano note onset detection method. In addition, a questionnaire survey is conducted on preschool children and their parents to investigate the popularization and teaching effect feedback of intelligent piano, which can provide a practical basis for the education and promotion of intelligent piano. The fourth section discusses the performance of the CNN model and the survey results. The final section summarizes the findings, reflects on inadequacies, and proposes prospects of the research work.

## Related Works

In the field of intelligent music teaching, automatic music transcription mainly focuses on the transcription of audio into symbolic music notation. However, traditional audio-only transcription methods are often affected by complex tuners and background noise. Sigtia et al. (2016) proposed an end-to-end DL framework, which could learn to automatically predict the initial event of notes under the given piano player video. Besides, this framework could transcribe and play music in the form of Musical Instrument Digital Interface (MIDI) data. Through experiments, the authors proved that this audio-combined video data transcription method could improve the quality of music transcribed from each mode alone. Li (2020) took piano composition as a sample to explore computer automatic composition and analyzed the application of DL and blockchain to the generation of digital music works. They reported that DL had great application potential in computer automatic composition of digital music, and the integration of blockchain and DL had played a role in promoting the expansion and popularization of the digital music market.

In the network era, piano teaching is facing unprecedented challenges and opportunities. Specifically, the piano teaching reform needs to innovate the curriculum system and improve teaching content *via* the network. Shuo and Xiao (2019) constructed a piano note recognition algorithm to calculate the corresponding annotation file by establishing the training and testing waveform data according to the MIDI file and the corresponding piano audio file. The authors utilized this algorithm to recompile the multi-voice model to construct a multi-voice recognition system, which offered inspiration for the system model of intelligent network piano teaching. Liu and Huang (2021) proposed an intelligent piano teaching system based on a neural network. The system realized the overall function of piano playing, including MIDI signal extraction, neural network model construction, playing interface, five-line animation, and evaluation interface. Moreover, aiming at the difficulties in computer piano teaching, they designed a piano performance evaluation system based on the artificial neural network model.

In summary, scholars have put forward some intelligent schemes for piano performance and teaching. However, there lacks a thorough investigation of the piano note onset detection based on DL technology. Therefore, combined with the current needs of piano teaching and the theoretical basis of DL and AI, the automatic scoring algorithm of piano music is put forward to provide reference and support for computer-aided piano teaching.

## Materials and Methods

### Features and Functions of Intelligent Piano

Intelligent piano teaching regards students as the absolute center, and the teaching content exactly serves students. Students are allowed to participate indirectly in the teaching design. Integration is realized in stages such as before class, in class, and after class. In the teaching design before class, teachers can analyze the interests and learning pace of students through big data and fine-tune the sections in teaching according to the analysis report made by the machine. In music teaching, if robots can really understand the language, emotions, feelings, and even thinking of teachers, the music played and understood by machines even surpass the breadth and depth of human thinking. The AI technology can present a continuous analysis of learning results to teachers and students, including valuable reports on achievements, learning status of students, and learning attitude of teachers, as well as any errors in the learning process and biased understanding of learning contents of students.

The advantage of the intelligent piano is that it can complete the accompanying practice without the presence of teachers, implement communication on a 4K display screen, demonstrate music, and teach playing skills with animation. It realizes the synchronous development of teaching, practice, game, and sharing. Intelligent pianos have the following functions.

The first function is the smart music score module. The intelligent piano has an enormous music library, and all music is stored in a tablet computer, through which players can retrieve any musical composition they want. The smart music score module is connected with an intelligent piano, and it can turn pages automatically during playing, which is convenient for the playing of children. Music scores are presented on an ultra-high-definition display, and the large size screen can protect the eyesight of children. Moreover, four pages of music scores can be displayed on the screen at the same time to reduce the trouble of children turning pages of music scores. The music score can be slowly scrolled or presented in the form of a waterfall to attract the interest of children in learning piano.

The second function is the automatic performance module. In an intelligent piano, the automatic performance module can be connected to the Internet for interactive transmission, remote control, and recording performance (Sinclair, 2018). Users can play music through video. The intelligent piano will automatically play along with the performance of the children so that children can learn intuitively.

The third function is the AI training module. Speech recognition technology can assist children to use the standard language in the system for personal repeated practice until they can be proficient in smart interactive teaching (Ahn and Lee, 2016). The function of AI training includes two modes, namely, class and practice. In the class mode, children choose a particular piano composition and learn through voice prompts. The system will prompt the segments that need to be practiced based on the performance of children. The practice mode supports user-defined exercises, and the system can generate the evaluation report smartly assessing and correcting the practice performance of children. In this way, children and their parents can master the situation of piano practice at any time.

The fourth function is the smart conversion of piano sound and silent mode. The silent mode of intelligent pianos will not affect the normal rest of others during piano playing. In the process of practicing the piano, children can wear earphones, so that only they can hear the sound of the piano. At this time, the sound of the piano can be converted into the sound of the electronic piano (Zabardasti et al., 2016). Some intelligent pianos can convert both the electroacoustic piano and the timbre of more than 120 different instruments so that children can feel the different timbres of musical instruments at home. The silence and voice conversion characteristics of the intelligent piano can provide a great convenience for piano learners and can improve their interest, making intelligent piano favored by most piano learners.

**FIGURE 1:**
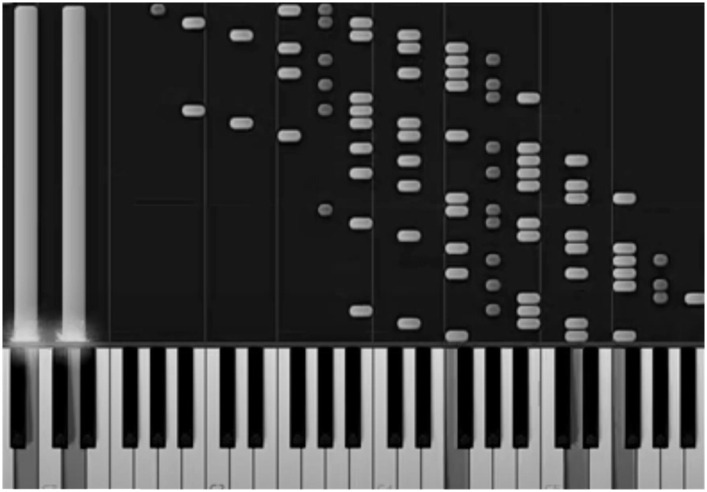
Waterfall flow music score. Free source image, available from https://www.shutterstock.com/.

The fifth function is the entertainment interaction module. In the era of IT, to stimulate the interest of children in learning piano to the greatest extent, researchers of intelligent piano software have added the section of a breakthrough game into the system. This game section enables most children to learn piano in a relaxed and pleasant learning environment, such as the “waterfall flow” game, as shown in Figure 1.

The sixth function is children can play any composition in the game. For most children, through the piano game mode, light emitting diode (LED) indicators can remind children to start practicing piano. Children can learn music in a relaxed and happy atmosphere, which can enhance their sensitivity to music, raise their interest in learning, and make piano learning no longer boring as in the traditional form. With the development of piano teaching applications, the intelligent piano can be connected to the teaching application software through smart devices to realize the interaction between people and the piano. For example, Dust Buster (a tool software) can adapt music scores into games to improve the interest of practice and enable children to have entertaining learning in their spare time. Furthermore, the network can help beginners practice the position of notes. The GarageBand application software can make music creation without using professional MIDI equipment but only using the intelligent piano connection, providing music lovers with as many conditions as possible.

The seventh function is the network piano classroom. There are two kinds of online piano classrooms *via* intelligent pianos. One is a piano classroom composed of multiple intelligent pianos, which requires teachers and students to complete piano teaching activities such as listening, playing, creating, and rehearsing, such as “The One—intelligent piano classroom,” “Find—intelligent piano classroom,” and “Pr—piano art studio.” The other is the network piano classroom, which can carry out intelligent piano distance education. Users can choose recorded or live video teaching courses according to their needs in the built-in system of intelligent pianos or the teaching program of the network piano classrooms. Moreover, teachers and students can interact with each other through network audio and videos. Teachers and students can not only communicate with each other through language and pictures but also use demonstration and other communication skills. Teachers can guide students according to their performance, which has the same effect as the field teaching mode. The teaching function of network piano classrooms can make the teaching method of an intelligent piano more flexible and intelligent.

The eighth function is smart evaluation. The evaluation function of intelligent pianos refers to the smart scoring according to the tune, rhythm, and strength of the performance of the students. Each student can understand their learning situation and shortcomings in each class. Smart evaluation for students is divided into three stages. First, for students who use the intelligent piano for the first time, it is necessary to assess their piano learning level, interest, and future expectations. The students without a piano foundation should learn the piano from the initial basic part, while the students with a piano foundation can choose a certain degree of difficulty to complete the test in intelligent piano. Second, it is necessary to score the tune, rhythm, and strength of the repertoire they can perform. Third, a learning plan suitable for their current stage is provided according to the scores of the students. The smart evaluation of the piano level of the students can help teachers teach students in accordance with their aptitude in a targeted way. The one-to-many group teaching mode is appropriate for elementary learners, while the one-on-one teaching mode is beneficial to high-level learners. In each stage of learning of the students, it is necessary to carry out smart evaluations of the learning situation of the students, which can motivate the learning enthusiasm of the students through group competition. Moreover, the smart evaluation module can send the music played by students and their achievements in each class to parents through the network, so that parents can know the learning situation of the students in time. Moreover, students can score their learning satisfaction by the self-evaluation method and complete the evaluation according to the opinions of the teacher. Teachers can master the cognition of the students of their learning and also make corresponding improvements and innovations in the teaching process according to the opinions of the students.

**FIGURE 2:**
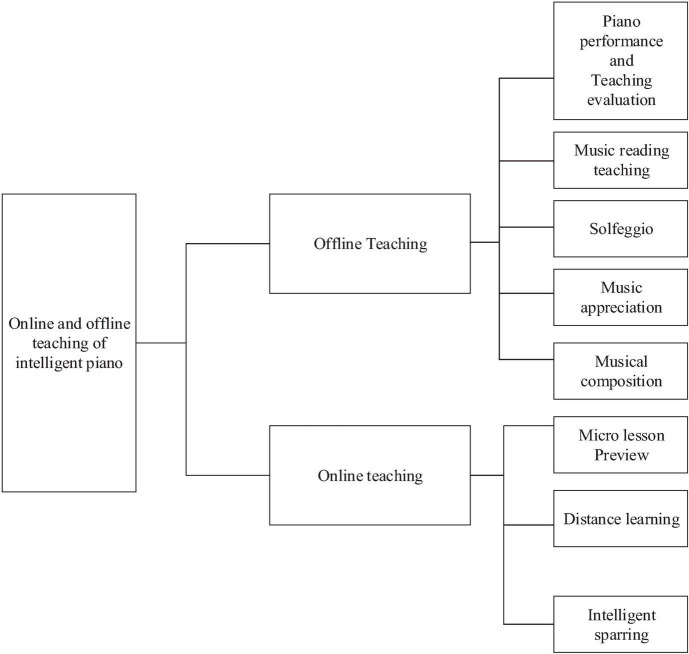
Basic teaching framework of the intelligent piano.

The ninth function is online and offline teaching modes. Offline teaching means that teachers and students complete face-to-face piano teaching in an intelligent piano classroom. Online teaching is complementary to offline teaching using intelligent piano micro-lecture videos, distance education, and smart partner training tools. Figure 2 reveals the primary teaching framework of intelligent piano constituted by online teaching and offline teaching.

Intelligent piano offline teaching refers to face-to-face teaching between the teachers and students in an intelligent piano classroom. Its teaching methods include piano performance, the teaching of music reading, solfeggio, music appreciation, and music composition. Preschool children must have a comprehensive study of piano enlightenment. Table 1 lists the features of intelligent piano offline teaching.

**TABLE 1:** Intelligent piano offline teaching.

Classification	Features
Piano performance	Teaching: teachers can utilize animation in intelligent piano software to assist teaching, so that obscure music theory knowledge is easy for learners to understand. Learning: children play the piano with the teacher. They can play separately, or the teacher can teach hand in hand. The difficulty increases gradually. The error correction function of intelligent piano can identify the errors of tune and rhythm in children’s performance, to improve the learning efficiency. Test: teachers guide children to play the piano in a scientific way, and monitor the playing process using the host computer. Teachers can conduct spot checks according to specific conditions, either one-to-one guidance or collective guidance. Evaluation: the teacher makes evaluation according to the children’s class situation, and selects the best player according to the scoring results of smart assessment and the specific score tips.
Teaching of music reading	Information technology is combined with teaching music reading. In the process of teaching, teachers use the resource base of intelligent pianos to establish a visual teaching situation, which can display music knowledge for preschool children in a more intuitive way and can accurately identify music scores.
Solfeggio	4–6 years old is the golden period of hearing development of preschool children. According to the effect of classroom teaching, teachers regard the game software of solfeggio as an auxiliary tool of classroom teaching. For example, listening games can improve preschool children’s ability to distinguish fixed notes, thus helping them learn solfeggio. Combining game teaching with intelligent piano can make children learn in the game and stimulate their learning enthusiasm.
Music appreciation	The teacher selects the micro-lecture video in the resource library. Children can feel different music and famous piano works. They listen to the story while listening to music and understanding music for a better experience of music.
Music composition	Children can use different rhythms and notes to create melodies. In the process of collective creation and discussion, preschool children can realize the significance of cooperation, and feel happy in music creation, which can improve their interest in music composition, and enhance children’s thirst for music knowledge.

Intelligent piano online teaching means that teachers use the Internet to teach piano, and learners can use smart functions in the learning process. The efficient use of intelligent piano micro-lecture videos, distance education, and smart partner training can supplement the shortcomings of offline teaching and promote the preview of children of new lessons and after-school practice, to enhance the learning efficiency of children. Table 2 illustrates the components of intelligent piano online teaching.

**TABLE 2:** The components of intelligent piano online teaching.

Component	Features
Micro-lecture video	According to the cognitive rules of preschool children, piano teaching using information technology is divided into fragmented learning and structured digital resources (short videos). Teaching activities are demonstrated by animation to fully mobilize preschool children to use various senses for learning, improve learning efficiency, and ultimately enhance the autonomous learning ability of children.
Distance education	The interconnection of intelligent pianos of the same type can realize remote transmission function. Teachers can fully understand the playing situation of children, and timely put forward the advantages and disadvantages of children in the process of playing. If the teacher and the child have different types of intelligent pianos, parents can transfer their children’s practice repertoire to the network platform and communicate with teachers in time. According to the children’s playing situation, the teacher teaches the children’s playing methods with the help of the remote guidance function of online teaching tools, to enhance the children’s playing ability.
Smart partner training	Intelligent piano teaching includes corresponding teaching software. Children can implement accompaniment exercises, distributed practice, and error correction. Once a child makes an error in the process of playing, the system will automatically reduce the playing speed, mark the wrong position on the spectrum surface, and remind with sound to prevent the same error next time. If the child plays correctly, encouraging sounds will be heard. The smart partner training function can make the children’s piano practice process rich in interesting operations, and greatly stimulate children’s interest in playing.

### Design of Piano Note Onset Detection Based on Deep Learning

**FIGURE 3:**
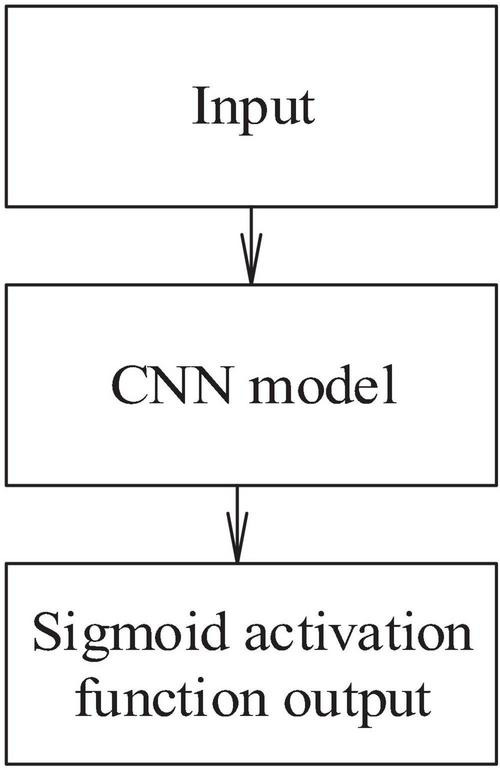
Piano music onset detection based on deep learning (DL).

From the perspective of music, the note onset is the starting point of a note in the piano score. From the perspective of the signal, the music signal is a continuous fluctuating wave. Besides, the note onset is the moment when the waveform envelope begins to change. Note onset detection is a fundamental topic in the field of content-based music information retrieval and the application of intelligent piano to piano teaching. Figure 3 displays the process of the piano music onset detection method based on DL reported here.

In the beginning, the time-frequency of the input piano music signal is analyzed, and the original time-domain waveform of the piano music signal is transformed into the frequency distribution varying with time. Here, short-time Fourier transform (STFT) is used to perform the time-frequency analysis (Khodja et al., 2019). The first step of this method is to read the audio file and reduce the value of signal sampling data to [−1, 1]. In the second step, if the audio is of multichannel type, the mean value of each channel data is taken to convert the sampled data into mono channel data. In the third step, “0” of half the frame length is filled in the beginning and end of the sampled data to eliminate sound before and after the recording. In the fourth step, the signal is truncated and divided into frames; then, the operation of windowing, fast Fourier transform, and amplitude spectrum calculation is performed, respectively. According to the pertinence of discrete Fourier transformation, the first half of the STFT amplitude spectrum of 88 piano notes is intercepted. Besides, the spectrum length is reduced from 2,049 dimensions to 512 dimensions to prevent the overfitting of the model.

**FIGURE 4:**
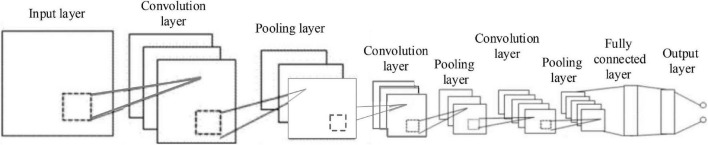
Convolution neural network (CNN) network structure.

Moreover, a CNN (Yao et al., 2020) model is designed for note onset detection. There is an input layer that is a continuous 9-frame Constant Q Transform (CQT) time-frequency representation, 4 convolution layers, 3 maximum pooling layers, and 2 fully connected layers, as presented in Figure 4.

Then, the convolution results of each convolution layer are batched standardly to speed up the training of the model and provide the regularization effect. Regarding the first fully connected layer, dropout is used with a probability of 0.5 to improve the generalization ability of the model. In addition, each output layer adopts the Rectified Linear Unit (ReLU) activation function. When the input is STFT time-frequency representation, the layer quantity and types of the CNN model remain unchanged, whereas the output dimensions and the parameters quantity of each layer change in accordance with the change in input dimension. Table 3 shows the parameter settings of the CNN model.

**TABLE 3:** Convolution neural network (CNN) network parameter setting.

Network layer	Parameter setting
Input layer	356*9
Convolutional layer	342*8*10@15*2 + Batch standardization + ReLU activation function
Pooling layer	171*8*10@2*2
Convolutional layer	160*6*20@12*3 + Batch standardization + ReLU activation function
Pooling layer	80*6*20@2*2
Convolutional layer	72*4*40@9*3 + Batch standardization + ReLU activation function
Pooling layer	36*2*40@2*2
Fully connected layer	256 + ReLU activation function + Dropout (*p* = 0.5)
Output layer	1 + sigmoid activation function

The training dataset of the CNN model is the MIDI Aligned Piano Sounds (MAPS), which is commonly used to evaluate the automatic recording task of piano music. Seven kinds of synthetic audios in the training set include complete piano music, as well as other synthetic audio, such as single notes and chords.

Finally, the onset of the note output by the CNN model is smoothed with the convolution of the 5 frames Hamming window. The corresponding time of the local maximum value higher than the given threshold is regarded as the onset of the note. Meanwhile, the onsets of several notes with time intervals less than 2 frames are regarded as one, and the average value is taken as the detection result.

### Application of Intelligent Piano to Piano Teaching for Children

**FIGURE 5:**
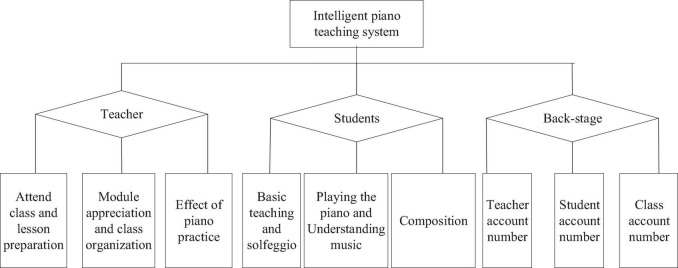
Intelligent piano teaching system.

The intelligent piano teaching system reported here works based on the Internet + teaching system (Ren et al., 2021), composed of the teacher teaching system, student learning system, and background management system, as illustrated in Figure 5.

Here, the piano music of *Jingle Bells* is used as a teaching example for intelligent piano teaching for preschool children. Teachers carry out intelligent piano teaching to help preschool children (aged 4–6 years) master music knowledge and the performance skills of the chord.

First, the psychological characteristics of preschool children aged 4–6 are expounded as follows. They have rich musical imagination and image memory, lively thinking, relatively distracted attention, and short concentration-time (Hua et al., 2015; Messerli-Bürgy et al., 2017). It requires teachers to develop appropriate teaching methods according to the rich imagination, curiosity, and creativity of preschool children, which can attract the attention of children to carry out complex teaching activities.

Second, the teacher uses the teaching function in the system to play the imported animation of piano lessons on the screen of the intelligent piano classroom. Then, the teacher uses multimedia to display the music, and children can mark the chord on the smart music score. The teacher explains the playing skills of a chord and demonstrates the correct performance. At the same time, the video of a standard performance is input into the system so that children can watch it repeatedly after class. Teachers also need to demonstrate the animation video of the fingering and explain the knowledge points according to the animation. In the process of playing, the teacher checks whether the hand shape and chord of each child are accurate and prompts fingering.

Finally, the children try to play *Jingle Bells*. In the one-to-many teaching mode, the teacher can ask the children to play in groups. In the process, the teacher checks the playing performance of each child and gives correct guidance.

### Feedback on the Intelligent Piano Teaching

The participants in the intelligent piano class are 40 preschool children aged 4–6 years. To ensure the authenticity of teaching feedback, a questionnaire survey is conducted for these preschool children and their parents.

## Results and Discussion

### Convolution Neural Network Model Performance

**FIGURE 6:**
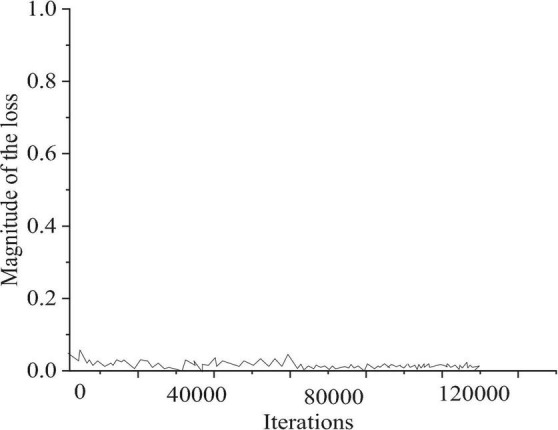
Loss curve of note onset detection in the network training process.

**FIGURE 7:**
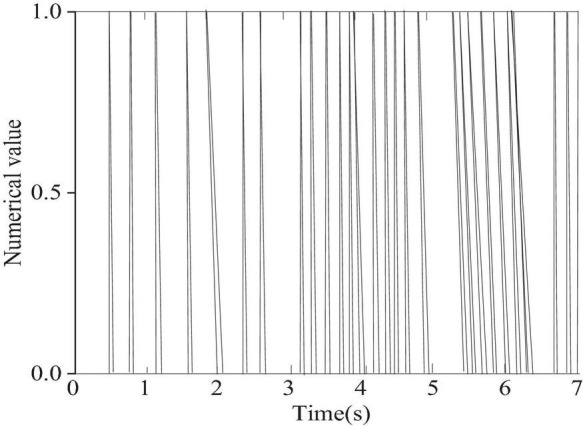
The result of onset detection is based on convolution neural network (CNN) network.

Figure 6 presents the loss during training of the CNN model using the training set, and Figure 7 provides the results of piano note onset detection of the model.

Figure 7 shows that as the training continues, the number of iterations gradually increases, and the loss value of the model in the training set gradually decreases. After 80,000 iterations, the loss value of the model finally tends to be stable, and the CNN model can realize the detection of the onset of the output note.

### Interest of Preschool Children in Learning Intelligent Piano

**FIGURE 8:**
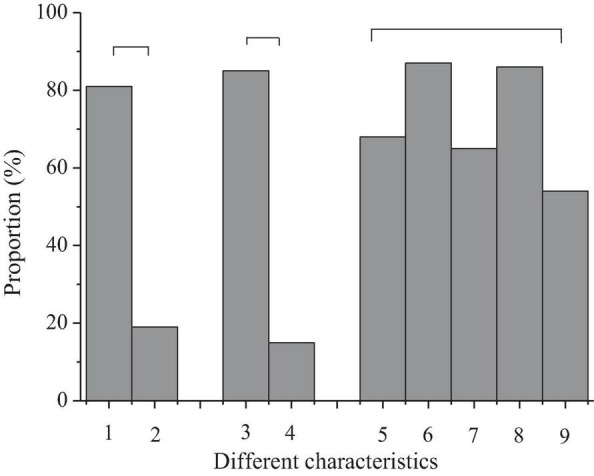
Interest of preschool children in learning the intelligent piano (“
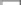
” represents the same dimensions; 1, children like intelligent music scores; 2, children like paper music scores; 3, children like piano lessons with games; 4, children do not like piano lessons with games; 5, children like reading music score; 6, children like playing the piano; 7, children like music appreciation; 8, children like music rhythm; 9, others).

According to the results of the questionnaire survey, the interest degree of 40 preschool children in the intelligent piano is shown in Figure 8.

Figure 8 shows that 80% of the children surveyed like an intelligent music score and 82% of them like an intelligent piano class with games. Moreover, piano playing is the most popular music activity for preschool children, followed by music rhythm, music score reading, and music appreciation.

The results indicate that preschool children have shown a strong interest in intelligent piano, which is consistent with the research results of Ying (2016). The interest of children in learning represents that preschool child treats learning with a positive attitude in the process of learning (Mills et al., 2017; Pappert et al., 2017). Intelligent piano teaching can stimulate the interest of preschool children and improve the efficiency of piano learning through its special teaching function and mode, which is worth popularizing.

### Intelligent Piano Learning Effect

**FIGURE 9:**
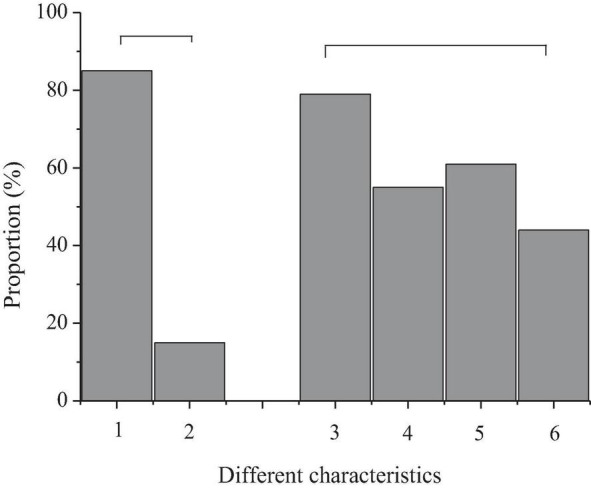
Learning effect of intelligent piano for preschool children (“
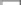
” represents the same dimensions; 1, children play new music actively; 2, children do not play new music actively; 3, intelligent piano has partner training function; 4, the intelligent piano can be used to teach remotely; 5, the intelligent piano has smart scores; 6, the intelligent piano has entertainment function).

Figure 9 illustrates the learning effect of 40 preschool children on intelligent piano.

Figure 9 shows that 84% of preschool children can take the initiative to learn in the process of intelligent piano teaching but 16% of children cannot. The partner training function of intelligent pianos can greatly help children to learn to play the piano. The other features with promotion function rank in order are smart music score, distance education, and entertainment function. In the field of piano performance, intelligent interaction primarily depends on the recording and analysis using electronic equipment. Therefore, the teaching concept of intelligent piano complements rational thinking in the teaching process and makes up for the limitations in the traditional piano teaching methods. The rapid interaction between students and teachers in teaching can be realized through the recording and feedback of digital equipment. This interaction avoids ineffective communication often caused by insufficient perception or comprehension of the music of students in the traditional teaching process.

Therefore, intelligent piano teaching can improve the enthusiasm of preschool children for practicing piano and develop their autonomous learning ability. In the intelligent piano class, preschool children can log in to their private accounts in the intelligent piano program according to the prompts of their parents and carry out the personal practice. Teachers can choose the partner training software mode (or the smart mode, independent practice mode, and original whole track speed competition) according to the situation of children (Bugos, 2018). The program can give a certain non-physical reward according to the proficiency of children in the repertoire, which plays a vital role in improving the self-confidence of children and reducing their fear of difficulties. Parents can let children practice by themselves, which can promote the development of independent habits of children and reduce the pressure of guidance of parents, and achieve win-win results. Intelligent piano teaching stimulates interest and efficiency of preschool children in learning piano through information management mode, situational teaching methods, game-based teaching process, and interactive teaching environment and makes children change from passive acceptance to active approaching to learning.

### Feedback of Parents on Intelligent Piano Courses

**FIGURE 10:**
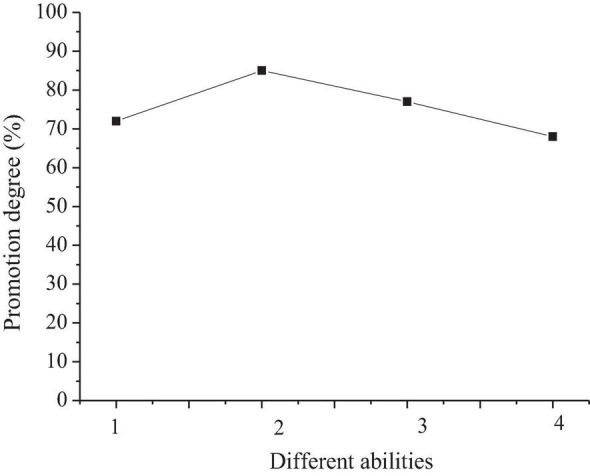
Feedback of parents on the intelligent piano course (1, the ability of children to read music; 2, the improvement of interest of children; 3, piano playing ability of children; 4, solfeggio ability of children).

According to the questionnaire results, the feedback of parents of 40 preschool children on the intelligent piano is displayed in Figure 10.

Figure 10 displays that 84% of parents of preschool children participating in intelligent piano teaching believe that the interest of their children in learning piano has increased. It demonstrates that parents are satisfied with the current teaching effect of the intelligent piano. Preschool children can learn intelligent piano with high enthusiasm, and their playing ability, music reading ability, and solfeggio ability have been greatly improved.

Teachers reasonably use the intelligent piano teaching mode to complete the teaching content of this course. Students have a deeper understanding and perception of the connotation of music and virtually put forward higher learning goals and requirements for themselves. Additionally, children in music rehearsal contact with each other more closely, and consequently, their cooperation ability, feelings, and friendship have been improved. Parents are the promoters of the learning of preschool children (Nimbalkar et al., 2016; Zhang et al., 2019). In the process of piano teaching, teachers play a leading role. However, if parents can accompany their children to learn piano every day, they can promote preschool children to learn piano happily. Intelligent piano teaching not only teaches the playing skills of children but also focuses on cultivating music comprehensive literacy and music creativity of children, which is also conducive to guiding children to establish a good network awareness.

## Conclusion

The AI technology and computer technology can assist piano teaching, through which learners can acquire music concepts and knowledge of western classical music, such as chromatic scale, transfer, interval, and chord. It can play a positive role in the teaching of the piano knowledge system. Based on the analysis of the functions and characteristics of intelligent piano, a method of detecting the onset of piano note is proposed based on CNN. Besides, intelligent piano teaching is introduced into a class of 40 preschool children aged 4–6 years to teach the piano lesson *Jingle Bells.* Finally, the learning interest and learning effect of learning feedback of the children and their parents are investigated through a questionnaire survey. The results show that CNN has good performance and can detect the onset of piano notes. Moreover, in the questionnaire survey, 80% of preschool children like smart music scores, 82% of preschool children like intelligent piano lessons with games, and 84% of preschool children have great enthusiasm for piano learning. Besides, 85% of parents think that the interest of their children in learning piano has increased. The findings prove that intelligent piano is loved by preschool children and their parents, which lays a good foundation for the smart development of enlightenment teaching of preschool children.

Due to limited personal ability and time, there are certain deficiencies. Although an automatic music transcription algorithm based on DL is constructed in this study, the accuracy of the algorithm needs to be further improved. In subsequent studies, the music language model will be used to learn common note combinations and note sequences. Based on these rules, the output of the acoustic model will be adjusted to solve the problem of fundamental frequency ambiguity. Moreover, the number of research objects and regional selection is insufficient. In a follow-up study, it is necessary to investigate cities of different consumption levels for an extensive sample size to improve the persuasiveness of the research results.

## Data Availability Statement

The raw data supporting the conclusions of this article will be made available by the authors, without undue reservation.

## Ethics Statement

The studies involving human participants were reviewed and approved by the Ethics Committee of Southwest University. The patients/participants provided their written informed consent to participate in this study. Written informed consent was obtained from the individual(s) for the publication of any potentially identifiable images or data included in this article.

## Author Contributions

The author confirms being the sole contributor of this work and has approved it for publication.

## Conflict of Interest

The author declares that the research was conducted in the absence of any commercial or financial relationships that could be construed as a potential conflict of interest.

## Publisher’s Note

All claims expressed in this article are solely those of the authors and do not necessarily represent those of their affiliated organizations, or those of the publisher, the editors and the reviewers. Any product that may be evaluated in this article, or claim that may be made by its manufacturer, is not guaranteed or endorsed by the publisher.
